# Effect of prolonged standardized bed rest on cystatin C and other markers of cardiovascular risk

**DOI:** 10.1186/1472-6793-11-17

**Published:** 2011-12-09

**Authors:** Karin Arinell, Kjeld Christensen, Stéphane Blanc, Anders Larsson, Ole Fröbert

**Affiliations:** 1 Department of Cardiology, Örebro University Hospital, Örebro, Sweden; 2 Institut Pluridisciplinaire Hubert Curien-De'partement d'Ecologie, Physiologie, Ethologie Unite' Mixte de Recherche 7178. Centre National de la Recherche Scientifique, Universite' de Strasbourg, Strasbourg, France; 3 Department of Medical Sciences, Clinical Chemistry, Uppsala University, Uppsala, Sweden

## Abstract

**Background:**

Sedentary lifestyle is associated with coronary artery disease but even shorter periods of physical inactivity may increase cardiovascular risk. Cystatin C is independently associated with cardiovascular disease and our objective was to investigate the relation between this novel biomarker and standardized bed rest. Research of immobilization physiology in humans is challenging because good biological models are in short supply. From the Women International Space simulation for Exploration study (WISE) we studied markers of atherosclerosis and kidney function, including cystatin C, in a standardized bed rest study on healthy volunteers. Fifteen healthy female volunteers participated in a 20-day ambulatory control period followed by 60 days of bed rest in head-down tilt position (-6°) 24 h a day, finalized by 20 days of recovery. The subjects were randomized into two groups during bed rest: a control group (n = 8) that remained physically inactive and an exercise group (n = 7) that participated in both supine resistance and aerobic exercise training.

**Results:**

Compared to baseline values there was a statistically significant increase in cystatin C in both groups after bed rest (P < 0.001). Glomerular filtration rate (GFR), calculated by both cystatin C and Cockcroft-Gault equation, decreased after bed rest while there were no differences in creatinine or creatine kinase levels. CRP did not change during bed rest in the exercise group, but there was an increase of CRP in the control group during recovery compared to both the baseline and the bed rest periods. The apo-B/apo-Ai ratio increased during bed rest and decreased again in the recovery period. Subjects experienced a small but statistically significant reduction in weight during bed rest and compared to baseline weights remained lower at day 8 of recovery.

**Conclusion:**

During and following prolonged standardized bed rest the concentrations of several clinically relevant cardiovascular risk markers change.

## Background

Sedentary lifestyle is associated with inflammation in population-based studies [1,2] and increases cardiovascular risk [3].

Bed rest causes muscle atrophy, which in turn leads to lower creatinine levels and decreased glomerular filtration rate (GFR), when calculated by the Modification of Diet in Renal Disease (MDRD) formula or Cockcroft-Gault formula dependant on creatinine [4]. Cystatin C is also a marker of GFR but is unaffected by muscle mass. However, age, sex, weight, smoking and high concentrations of CRP affect the plasma level of cystatin C [5]. Because bed rest and cystatin C levels are both cardiovascular risk factors we found it of interest to investigate how cystatin C, together with other risk markers, are affected by prolonged standardized bed rest.

It has been proposed that elevated cystatin C levels are directly correlated to both inflammation and atherosclerosis [6]. High cystatin C levels are independently associated with cardiovascular risk factors such as BMI, low HDL cholesterol and smoking even in patients without chronic kidney disease or microalbuminuria [7]. Cystatin C is independently associated with cardiovascular disease after adjustment for major cardiovascular risk factors [8]. Jernberg et al. demonstrated an association between cystatin C level and mortality in patients with suspected or confirmed non-ST-elevation acute coronary syndrome [9].

Research of immobilisation and human physiology is challenging because good biological models are in short supply. Interpretation of biological samples in hospitalized patients may be biased by various disease states, surgical and other invasive procedures and non-standardized bed rest regimens. In collaboration with the Women International Space simulation for Exploration study (WISE) conducted in 2005 [10] we studied kidney function and atherosclerosis/inflammation markers in a two-month bed rest study on healthy volunteers enabling research on the physiopathology of immobilization.

## Methods

### Study design

Sixteen healthy female volunteers participated in a 60-day bed rest study. All were non-smokers, free of any clinical/biomedical sicknesses and had not taken any contraceptive pills 3 months prior to the study. An additional inclusion criterion was that the participants were required to exercise 30 minutes per day (moderate activity as structured exercise or activities in daily living) prior to the study.

**Figure 1:**
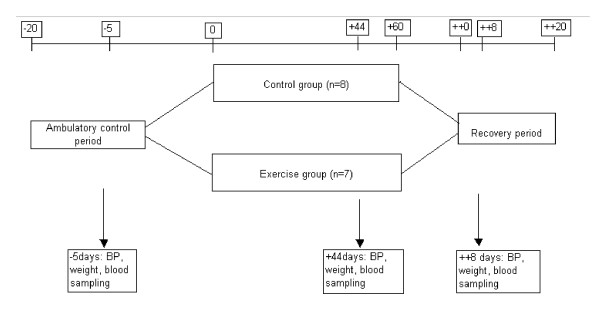
**Overview of the bed rest study**. A 20-day ambulatory control period was followed by 60 days of bed rest in head-down tilt position (-6°) and the study was finalised by 20 days recovery period.

A 20-day ambulatory control period was followed by 60 days of bed rest in head-down tilt position (-6°) 24 h a day and the study was finalised by a 20 day recovery period (Figure 1). Baseline data collection was performed in the ambulatory control period. The subjects were randomized into two groups (n = 8, each) during bed rest: a control group that remained physically inactive and an exercise group that participated in both supine resistance and aerobic exercise training. Fifteen of the participants gave informed consent to participate in our study.

The resistance training included 19 sessions of 45 minutes training on a flywheel ergometer (including 10 min of warm up). The aerobic training was designed as 29 sessions using a specially designed vertical treadmill. Each session lasted 50 ± 2 min at varying intensities between 40 and 80% of pre-bed rest maximum oxygen uptake. All sessions were equally distributed during the entire bed rest period. The detailed protocol of the WISE experiment and the training sessions are described in detail in previous reports [10].

### Laboratory analysis

Blood samples were collected in heparin-containing tubes at baseline (five days before bed rest), after 44 days of bed rest and 8 days into the recovery period (Figure 1). All samples were taken within 15 min after breakfast. The samples were centrifuged immediately at 3000 rpm for 10 min, and plasma was frozen within 30 min and stored in aliquots at -80°C.

Plasma apolipoprotein A1 (reagent: 9D92-01), apolipoprotein B (reagent: 9D92-01), creatine kinase (reagent: 7D63-20), creatinine (reagent: 8L24-01), C-reactive protein (reagent: 6K2601) and cystatin C (Cyc-C) (reagent: 1014; Gentian, Moss, Norway) were analyzed on an Architect Ci8200 (Abbott Laboratories, Abbott Park, IL, USA) and reported using SI units. The total analytical imprecision of the assays were: 1.8% at 0.68 g/L and 1.2% at 2.0 g/L for apolipoprotein A, 4.5% at 0.47 g/L and 2.4% at 1.68 g/L for apolipoprotein A, 0.7% at 2.8 μkat/L and 0.8% at 13 μkat/L for creatine kinase, 4.8% at 70 mmol/L and 4.8% at 94 mmol/L for creatinine, 0.8% at 8 mg/L for C-reactive protein and 1.7% at 0.77 mg/L and 1.1% at 1.25 mg/L for cystatin C.

GFR was calculated using the Cockcroft-Gault formula, which estimates GFR in mL/min.

$$\text{GFR}=\frac{\left(140-\text{Age}\right)\times \text{Mass}\left(\text{in kilograms}\right)\times \text{constant}}{\text{Serum creatinine }\left(\text{in micromol}∕\text{L}\right)},$$

where *constant *is *1.23 *for men and *1.04 *for women.

We used a cystatin C immunoassay from Gentian (Gentian, Moss, Norway) on Architect ci8200 (Abbott Laboratories, Abbott Park, Ill., USA) to calculate GFR in mL/min/1.73 m^2^. The formula for calculating GFR with cystatin C is eGFR (mL/min/1.73 m2) = 79.901* (cystatin C value in mg/L) ^-1.4389 ^[11].

### Statistical analysis

Statistical analysis was performed using SigmaStat 3.5 software (Systat, San Jose, Ca). Blood sample results were compared by a two way repeated measures analysis of variance and a p-value of < 0.05 was considered statistically significant.

## Results

Baseline characteristics of the volunteers: age, height, weight, body mass index (BMI) and blood pressure are summarized in table 1. Weight decreased in both groups after 44 days of bed rest and the weight reduction was still statistically significant in the recovery period when compared to baseline. After 8 days of bed rest there was a small but significant weight increase in the control group, but not in the exercise group, compared to the bed rest period.

**Table 1:** Baseline data of age, height, weight, BMI, systolic and diastolic blood pressure.

	Baseline		After 44 d of bed rest		After 8 days of recovery	
	
	Control	Exercise	Control	Exercise	Control	Exercise
N	8	7	8	7	8	7
Age (yr)	34 ± 4	34 ± 3	34 ± 4	34 ± 3	34 ± 4	34 ± 3
Height (m)	1.63 ± 0.06	1.67 ± 0.05	1.63 ± 0.06	1.67 ± 0.05	1.63 ± 0.06	1.67 ± 0.05
Weight (kg)	55.6 ± 3.9	59.3 ± 2.7	52.6 ± 4.0 *#	56.5 ± 5.7 *	53.6 ± 4.2 *	56.4 ± 4.8 *
BMI (kg/m^2^)	21 ± 1.2	21.3 ± 1.6	19.9 ± 1.2 *#	20.4 ± 1.6 *	20.2 ± 1.3 *	20.3 ± 1.6 *
Systolic blood pressure (mmHg)	97 ± 8	96 ± 8	98 ± 11	104 ± 10	96 ± 14	101 ± 8
Diastolic blood pressure (mmHg)	59 ± 6	55 ± 8	63 ± 11 *	64 ± 7 *	60 ± 13	60 ± 10

Weight decreased during bed rest in both groups. The exercise group had not gained weight 8 days into the recovery period, but the control group gained some weight.

* = statistically significant difference compared to baseline

# = statistically significant difference compared to recovery period

There was no statistically significant difference between the groups.

**Figure 2:**
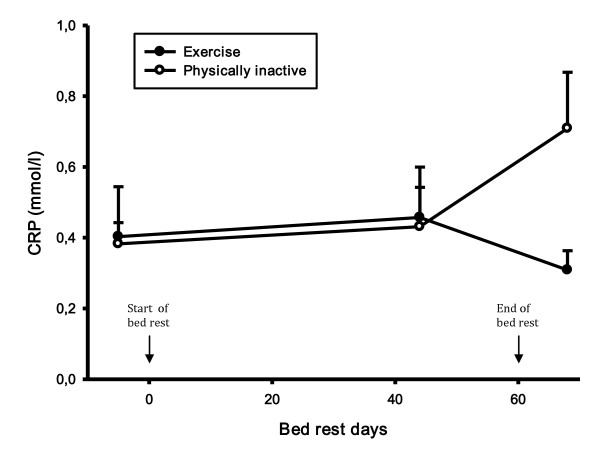
**CRP levels during bed rest study**. CRP did not change during the bed rest period in the exercise group, but there was a statistically significant increase in CRP in the control group in the recovery period compared to both the control period and the bed rest period.

**Figure 3:**
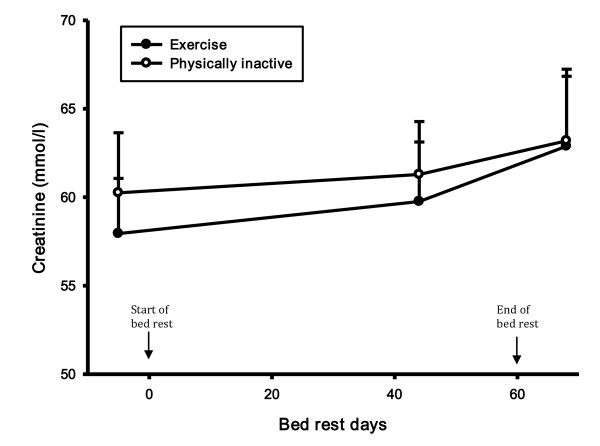
**Creatinine levels during bed rest study**. There was no difference in creatinine levels.

**Figure 4:**
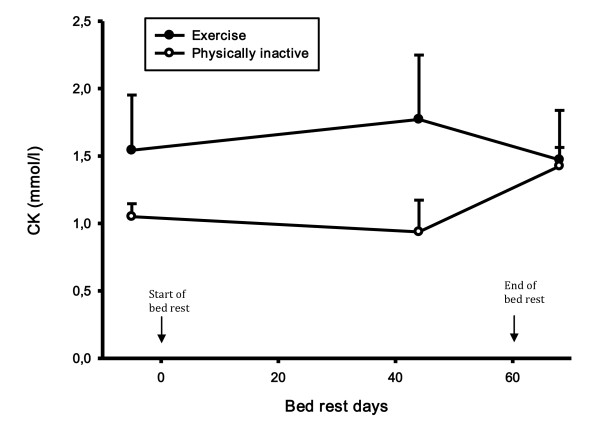
**Creatine kinase levels during bed rest study**. There was no difference in creatine kinase levels.

**Figure 5:**
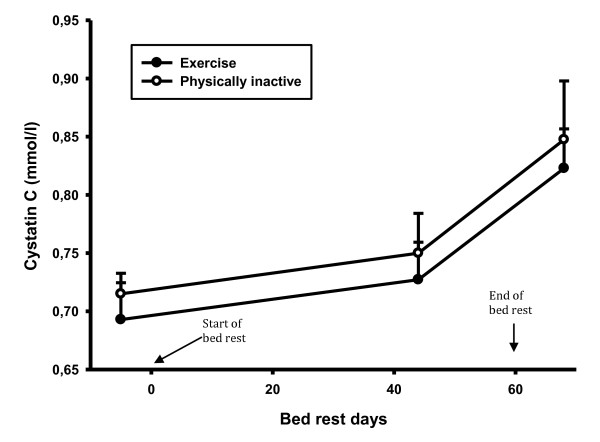
**Cystatin C levels during bed rest study**. There was a statistically significant increase in cystatin C after the bed rest completion.

**Figure 6:**
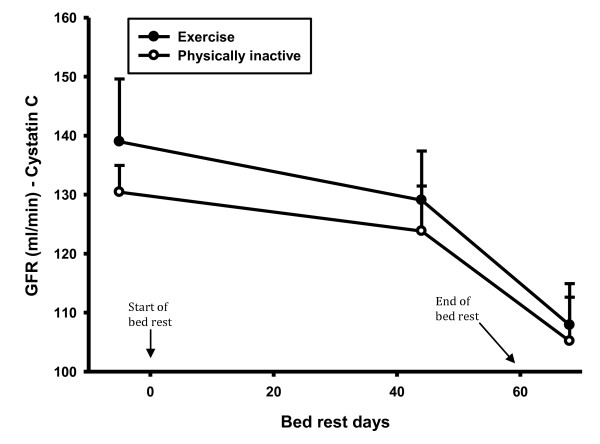
**GFR levels during bed rest study**. There was a statistically significant decrease in GFR, calculated with cystatin C in both groups after the bed rest completion.

**Figure 7:**
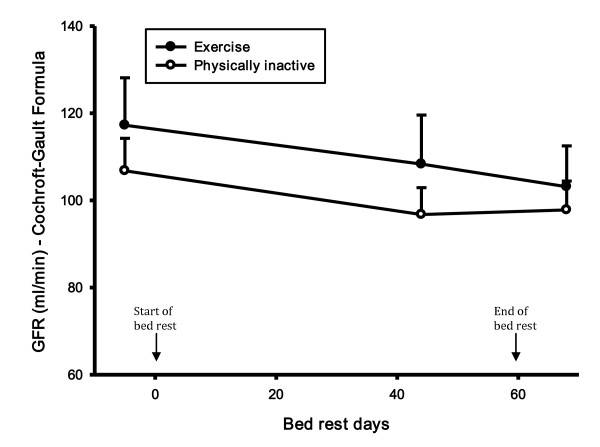
**GFR levels during bed rest study**. There was a statistically significant decrease in GFR, calculated with the Cockcroft-Gault formula in both groups after the bed rest completion.

CRP did not change during the bed rest period in the exercise group, but there was a statistically significant increase in CRP in the control group in the recovery period compared to both the control period (46%, p-value 0.008) and the bed rest period (39%, p-value 0.021) (Figure 2). During the recovery period there was a statistically significant difference in CRP between groups (56%, p-value 0.025). There was no difference in creatinine and creatine kinase levels (Figure 3 and 4). There was a statistically significant increase in cystatin C in both groups 8 days after bed rest compared to baseline (16%, p-value < 0.0001) and 44 days of bed rest (12%, p-value < 0.0004) (Figure 5). GFR calculated with cystatin C decreased significantly in both groups after bed rest completion (Figure 6) compared to baseline (26% decrease, p-value < 0.0001) and 44 days of bed rest (19%decrease, p-value 0.0002). Similarly GFR calculated with the Cockcroft-Gault formula decreased after bed rest completion (Figure 7) compared to baseline (11%, p-value 0.001) and 44 days of bed rest (2%, p-value 0.005).

**Figure 8:**
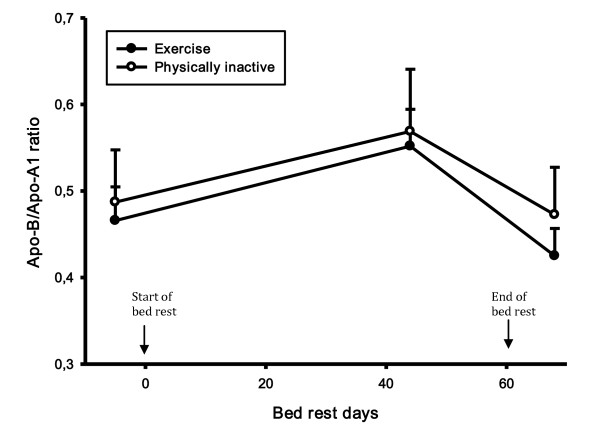
**Lipid levels during bed rest study**. The apo-B/apo-Ai ratio increased after 44 days of bed rest and decreased again 8 days into the recovery period.

The apo-B/apo-Ai ratio increased after 44 days of bed rest (15%, p-value < 0.0001) and decreased again 8 days into the recovery period (25%, p-value < 0.0001) (Figure 8).

## Discussion

In this study we measured the effect of bed rest on plasma concentrations of cystatin C, which is an emerging marker of cardiovascular disease. We also measured traditional markers of inflammation and kidney function. Eight days after a bed rest period of 60 days was completed cystatin C levels increased and GFR decreased and for GFR this was independent of calculation method.

Bed rest is probably the single most applied therapy for hospitalized patients no matter the underlying disease. It is therefore of great importance to understand the pathophysiological changes during immobilization as it may influence treatment. The growing problem of "sedentary lifestyle" could also be considered as a global immobilisation experiment. Future trips to Mars require further information of the effect of immobilization to the human body.

Patients with elevated levels of cystatin C are at higher risk of developing cardiovascular diseases [8,9]. Low glomerular filtration rate is a risk factor for cardiovascular mortality, independent of other cardiovascular risk factors [12]. Peralta el al discussed the probability that cystatin C might be a better parameter for identifying patients with chronic kidney disease at risk of developing cardiovascular complications than a creatinine-based equation [13]. Other researchers found that elderly people with the highest quintile of cystatin C (1.29 mg/i) have a significantly elevated risk of death from cardiovascular causes, myocardial infarction, and stroke after multivariate adjustment [14]. Cystatin C is linearly associated with cardiovascular mortality, but creatinine on the other hand predicts worse outcome only in patients with severe kidney dysfunction [15].

It has been described that high cystatin C levels correlate to an extensively increased risk of cardiovascular events in persons who do not meet the criterion of eGRF ≤ 60 mL/min/1.73 m2, a definition of chronic kidney disease [16]. High cystatin C levels have been found to be associated with elevated levels of CRP and [5,17] and other inflammatory markers such as IL-6, tumour necrosis factor alpha (TNF-α), and two soluble TNF-α receptors, even with creatinine-based eGFR ≥ 60 mL/min/1.73 m2 [18]. How non-renal factors influence cystatin C concentrations need further research.

In our study CRP was elevated after bed rest in the non-exercise group. Our finding might reflect that bed rest increases inflammatory activity, which in turn advances the atherosclerotic process leading to enhanced risk of CVD as well as elevated cystatin C levels by atherosclerosis in the kidneys and thereby a decreased glomerular filtration rate. In a recent study of overweight/obese postmenopausal women practising physical activity, it was shown that women with the highest tertile of physical activity energy expenditure had lower concentrations of hsCRP after adjustment for fat mass [19]. This can give an explanation to the difference in CRP between the control group and the exercise group in our study.

Cystatin C also has a different role in relation to inflammation/atherosclerosis. Inflammatory cytokines associated with atherosclerosis stimulate the production of lysosomal cathepsins and increase the plasma concentration of cystatin C. Cystatin C is a cathepsin inhibitor and might therefore play a roll in counterbalancing a potentially destructive greater elastolytic activity [20]. Mice deficient in cystatin C have increased elastic lamina degradation and greater atherosclerotic plaque formation. Studies have shown that both cathepsins and their inhibitor cystatin C could act either pro- or antiatherogenic in the different stages of atherosclerosis [21]. This role of cystatin C probably plays a lesser part in our study.

A limitation to our study is that the blood samples were taken in 2005 and analysed five years later. This might have influenced our results. A recent study however, has shown that when using the Gentian method the cystatin C levels were stable when comparing blood samples over four years of time [22].

All samples were analyzed in a single batch and in a random mode with the same reagent batch and the same calibration on a single instrument. The instrument has a high assay capacity so the time interval between first and last assay were less than 30 min. This in combination with the low assay CVs makes it unlikely that the differences in this study are due to variation in the assay or sample evaporation during the assay. Our findings were done in women only and the groups were relatively small. Knight et al. found in a cross-sectional study that male gender was independently associated with higher serum cystatin C after adjusting for creatinine clearance. Older age, greater height and weight have a similar effect. Previously, serum cystatin C levels have been found to correlate somewhat with weight [5]. In our study there was a statistically significant weight reduction in both groups after 44 days of bed rest and in the recovery period compared to baseline. According to Knight et al. because of weight loss a reduction in cystatin C levels could have been expected. In vertebrates both metabolic rate and glomerular filtration rate are positively correlated to body size [23]. However, we found the opposite - an increase in cystatin C levels. This could indicate that cystatin C is a marker of other physiological processes than kidney function. Knight et al. showed that high CRP levels are independently associated with increased cystatin C levels after adjustment for creatinine clearance. Cystatin C can also be a biomarker for inflammation [24,25]. In another substudy of WISE it was shown that bed rest causes both mechanistic and functional impairment of endothelial function [26]. These results could serve as a possible explanation for the cystatin C findings in our study - because early changes in endothelial function are part of the pathogenesis of atherosclerosis.

The decrease in GFR calculated with the Cockcroft-Gault formula can be explained by a combination of weight reduction and stable creatinine values. Weight reduction in the control group was most likely due to muscle atrophy and stable fat mass. Unexpectedly, Bergouignan et al. showed that the desire to eat was reduced in the exercise group - leading to a negative energy balance and a decrease in fat mass [27]. It is a limitation to our study that a golden standard measurement, as e.g. iohexol clearance, was not used. We did, however, use two different methods to calculate GFR, both dependent (the Cockcroft-Gault formula) and independent of weight (cystatin C) and the findings were identical - a decrease in GFR after bed rest. In a recent cross sectional study of persons with early stages of chronic kidney disease, light and total physical activity were positively correlated to kidney function when measured by MDRD. This relationship lost statistical significance after adjustment for BMI, cholesterol, CRP and mean arterial blood pressure. Increased physical activity may reduce the progression of chronic kidney disease by decreasing oxidative stress and inflammation and reducing blood pressure besides the positive effect of weight loss [28]. Conceivably decreased physical activity, in our study bed rest, has the opposite effect with an increase in oxidative stress and inflammation.

We cannot explain why the changes were seen first *after *the bed rest study was completed and not *during *bed rest. The effect could be due to the change from resting to standing position as it has previously been shown that body position per se influences renal perfusion [29]. Further studies are required to determine the mechanism of the effect of bed rest on cystatin C as well as other cardiovascular risk markers.

## Conclusion

In conclusion, cystatin C increased and cystatin C estimated GFR decreased in healthy female volunteers after a standardized bed rest period of 60 days. As cystatin C is a cardiovascular risk marker, this study may implicate that longer time periods of immobilization augment the risk of atherosclerosis. This is however only a hypothesis. Further studies are warranted to explore the role of cystatin C as a link between inactivity and cardiovascular risk.

## Competing interests

The authors declare that they have no competing interests.

## Authors' contributions

KA, KC, AL and OF were involved in the conception and design of this project. SB contributed with acquisition of data. KA and OF analysed data. KA, KC and AL were responsible for interpretation of data. The manuscript was drafted by KA and all other authors revised it critically. All approved of publication.
